# Effect of antimicrobial administration on fecal microbiota of critically ill dogs: dynamics of antimicrobial resistance over time

**DOI:** 10.1186/s42523-022-00178-9

**Published:** 2022-06-04

**Authors:** Julie Menard, Robert Goggs, Patrick Mitchell, Yufan Yang, Sarah Robbins, Rebecca J. Franklin-Guild, Anil J. Thachil, Craig Altier, Renee Anderson, Gregory G. Putzel, Holly McQueary, Laura B. Goodman

**Affiliations:** 1 Department of Veterinary Diagnostic and Clinical Sciences, Faculty of Veterinary Medicine, University of Calgary, Calgary, AB Canada; 2 Department of Clinical Sciences, College of Veterinary Medicine, Cornell University, Ithaca, NY USA; 3 Department of Population Medicine and Diagnostic Sciences, College of Veterinary Medicine, Cornell University, Ithaca, NY USA; 4 Microbiome Core Lab and Jill Roberts IBD Institute, Weill Cornell Medicine, Cornell University, New York City, NY USA; 5 Department of Public and Ecosystem Health, College of Veterinary Medicine, Cornell University, Ithaca, NY USA

**Keywords:** Antimicrobial resistance genes, Veterinary, Sepsis, Microbiota, *Escherichia coli*, *Enterococcus*

## Abstract

**Background:**

Multidrug resistance in companion animals poses significant risks to animal and human health. Prolonged antimicrobial drug (AMD) treatment in animals is a potential source of selection pressure for antimicrobial resistance (AMR) including in the gastrointestinal microbiota. We performed a prospective study of dogs treated for septic peritonitis, pyometra, or bacterial pneumonia and collected repeated fecal samples over 60 days. Bacterial cultures and direct molecular analyses of fecal samples were performed including targeted resistance gene profiling.

**Results:**

Resistant *Escherichia coli* increased after 1 week of treatment (D1:21.4% vs. D7:67.9% *P* < 0.001) and returned to baseline proportions by D60 (D7:67.9% vs D60:42.9%, *P* = 0.04). Dogs with septic peritonitis were hospitalized significantly longer than those with pneumonia or pyometra. Based on genetic analysis, Simpson’s diversity index significantly decreased after 1 week of treatment (D1 to D7, *P* = 0.008), followed by a gradual increase to day 60 (D1 and D60, *P* = 0.4). Detection of CTX-M was associated with phenotypic resistance to third-generation cephalosporins in *E. coli* (OR 12.1, 3.3–68.0, *P* < 0.001). Lincosamide and macrolide-resistance genes were more frequently recovered on days 14 and 28 compared to day 1 (*P* = 0.002 and *P* = 0.004 respectively).

**Conclusion:**

AMR was associated with prescribed drugs but also developed against AMDs not administered during the study. Companion animals may be reservoirs of zoonotic multidrug resistant pathogens, suggesting that veterinary AMD stewardship and surveillance efforts should be prioritized.

**Graphical abstract:**

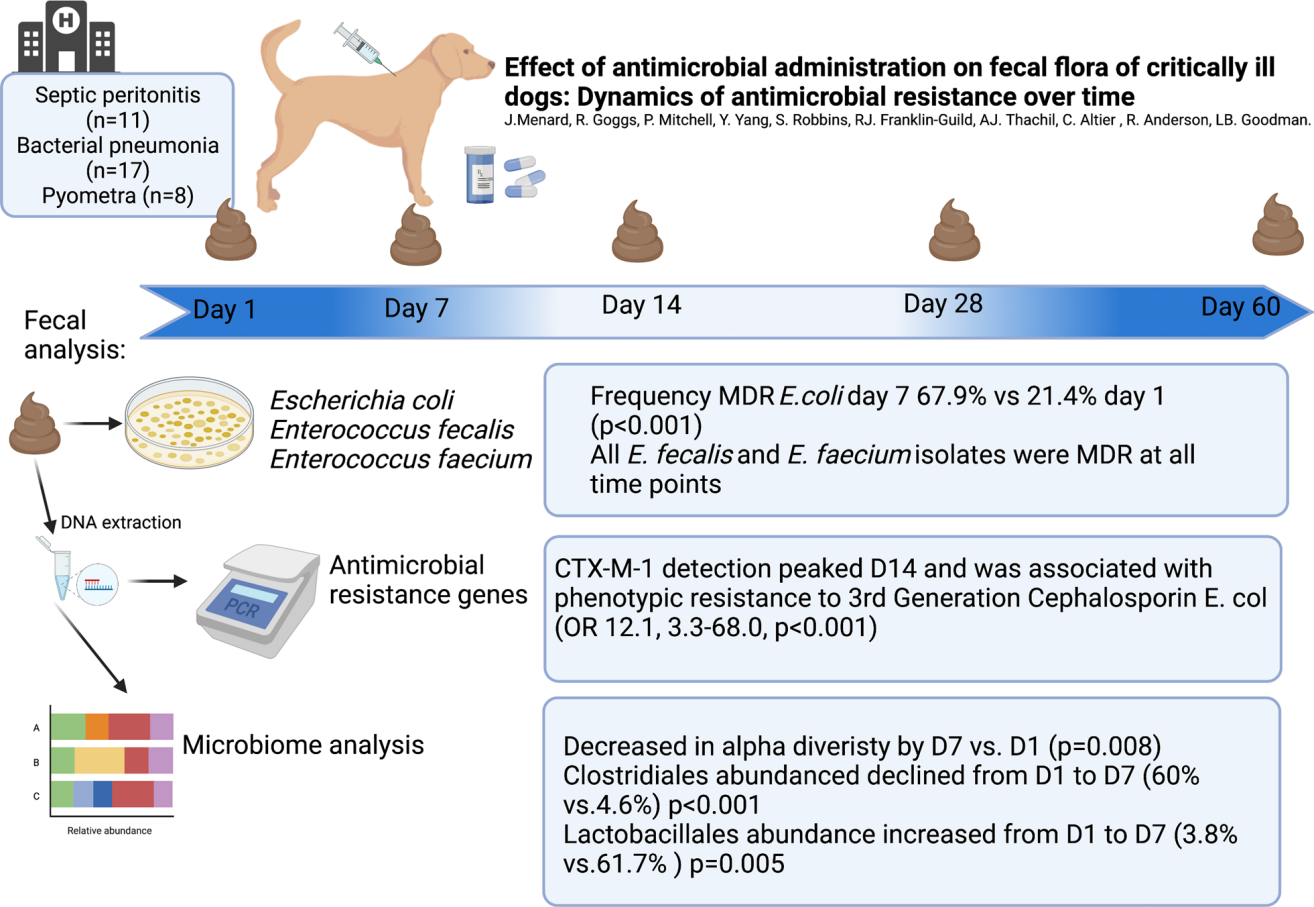

**Supplementary Information:**

The online version contains supplementary material available at 10.1186/s42523-022-00178-9.

## Background

The gastrointestinal microbiome is a complex ecosystem of multiple coexistent bacterial species that compete for space and nutrients [1], thereby minimizing colonization by pathogens through immune regulation and by outcompeting them for resources [2]. Sepsis is a life-threatening syndrome associated with mortality rates of 22–68% in dogs [3, 4]. Treatment consists of administration of empirical, broad-spectrum, antimicrobial drugs (AMDs) followed by de-escalation based on susceptibility testing, but many dogs receive AMDs for 4–6 weeks based on textbook recommendations [5, 6]. Prolonged AMD treatment has myriad effects on the gastrointestinal microbiome, altering the constituents of the microbiota, causing dysbiosis and exerting a selection pressure favoring emergence of resistant pathogens [7]. Antimicrobial resistance (AMR) genes in some gastrointestinal bacteria constitute the resistome; a reservoir of resistance genes transmissible through horizontal gene transfer to other gastrointestinal microbiota. Bacterial AMR can develop within days of initiating AMD treatment and commonly resolves soon after discontinuation [8–10]. Consistent with the presence of a gastrointestinal microbiota resistome, fecal *Escherichia coli* in dogs can rapidly develop AMR against AMDs to which they were not exposed [11]. Most studies have focused on the impact of AMD administration on fecal *E. coli* and *Enterococcus* spp. with the two organisms considered representative of the Enterobacterales and Gram-positive intestinal aerobes, respectively [8–11]. Molecular techniques enable evaluation of the entire gastrointestinal microbiota and assessment of population shifts in response to disease and AMD administration. In vitro resistance assessments are also needed for veterinary infections for which genotype: phenotype correlations are less well established. Changes within specific bacterial genera or in the ratios of bacterial classes are associated with prolonged hospital stay, bacteremia and sepsis in humans [12–14]. Alterations in the human gastrointestinal microbiome might promote multiple organ dysfunction syndrome, although the mechanisms remain indistinct [15]. The gastrointestinal microbiomes of humans and dogs are similar [16], suggesting that phenomena observed in humans might also affect dogs [1, 17]. Gastrointestinal dysbiosis occurs in dogs with exocrine pancreatic insufficiency, inflammatory bowel disease, intestinal lymphoma and parvoviral enteritis [18–21], but the impact of AMD administration on the fecal microbiota of dogs with bacterial sepsis has not been studied. We aimed to determine the effects of AMD administration to critically ill dogs on the emergence of phenotypic resistance by culture and susceptibility testing of fecal *E. coli*, *E. faecium* and *E. faecalis*; describe the impact of AMD treatment on AMR gene acquisition within the fecal microbiota using quantitative PCR; and describe changes in fecal microbiota diversity in response to AMD treatment using 16S rRNA gene sequencing. It was hypothesized that AMR in enteric sentinel bacteria increases during AMD treatment and resolves following drug discontinuation; enteric bacteria acquire genes conferring resistance to AMDs administered and those not administered; and that fecal microbiota diversity decreases during AMD administration and increases after drug discontinuation.

## Results

### Patient population

**Fig. 1:**
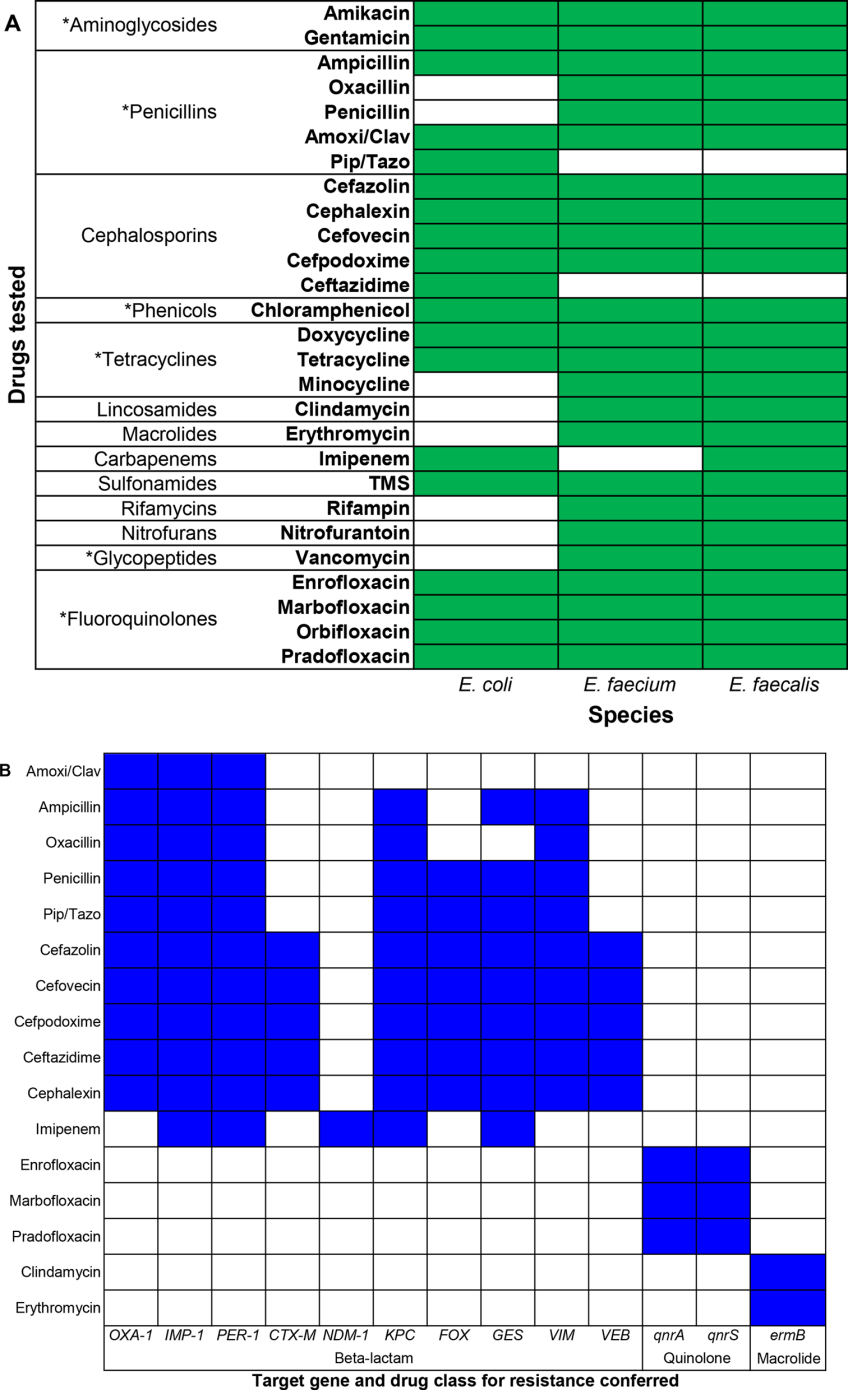
**A** Drugs tested (with antimicrobial class and drug displayed on the y axis) and microbiological susceptibility results for *Escherichia coli*, *Enterococcus faecium* and *Enterococcus faecalis* are shown in green color on the x axis. *Indicates antimicrobial class used to determine multidrug resistant status defined as resistance to ≥ 1 agent in ≥ 3 antimicrobial categories per Magiorakos et al. [27]. *Amoxi/Clav* Amoxicillin/clavulanate; *Pip/Tazo* Piperacillin/Tazobactam; *TMS* trimethoprim/sulfamethoxazole. **B** ABRx Panel results (Diatherix-Eurofins, Hunstville, AL): 17 antimicrobial resistance genes and corresponding drugs (shown in blue) to which bacteria would show resistance if gene is present

A total of 61 dogs were enrolled; 14 dogs were subsequently withdrawn and a further 10 dogs were euthanized (poor prognosis and financial limitations) within the first 7 days. Of the 37 dogs remaining at D7, 1 was euthanized (diagnosed with CNS lymphoma) on day 18, and 1 dog was euthanized on day 28 due to disease recurrence. The overall mortality rate was 19.7% (12/61), with 35 dogs completing the study to D60 (Additional file 1: Figure S1). Analyses were conducted for 36 dogs with samples available on D1, D7 and D14. Of these 36 dogs, 17 had bacterial pneumonia (4 mixed breed, 2 Great Dane, 2 German shepherd, and one of each of the following: Rottweiler, St Bernard, Cairn Terrier, French Bulldog, Border Collie, Beagle Hound, West Highland White Terrier, Newfoundland, Labrador Retriever); 11 had septic peritonitis (3 Labrador Retriever, 3 mixed breed, and one of each of the following: St Bernard, Great Pyrenees, Mastiff, American Eskimo, Siberian Husky); and 8 had pyometra (4 mixed breed, 2 Siberian Husky, 1 German Shorthaired Pointer and 1 Basset Hound). Descriptive statistics for all dogs are summarized in Table 1. Dogs with pyometra were older than dogs with septic peritonitis (*P* = 0.01) and pneumonia (*P* = 0.002). Illness severity scores (APPLE_fast_) in dogs with septic peritonitis or pyometra were greater than dogs with pneumonia (both *P* = 0.017). Dogs with septic peritonitis were hospitalized for longer than dogs with pneumonia (*P* = 0.02) or pyometra (*P* = 0.018). In the overall population, illness severity scores were positively correlated with duration of hospitalization (ρ 0.35, *P* = 0.04), but these correlations were not significant within groups (Fig. 1).

**Table 1:** Patient population description, with median and IQR

Value median (IQR)	Septic peritonitis (n = 11)	Pyometra (n = 8)	Bacterial pneumonia (n = 17)	All dogs (n = 36)
Age (years)	1 (1–7.5)	10.5 (9.75–11.25)*	2.4 (1–6.8)	6 (1–9.25)
Body weight (kg)	25.90 (18.45–42.25)	32.70 (27.52–36.88)	23.00 (12.70–43.00)	27.05 (16.32–40.10)
Sex (F/FS/M/ MN)	2/5/3/1	8/0/0/0	1/7/6/3	11/12/8/5
APPLE_fast_ score	25.5 (20.75–29.5)^#^	21.5 (20–25.5)	14.5 (12.5–18)*^#^	20 (14–25.75)
Length of hospitalization (days)	5.5 (4–7.5)*	2.75 (2.5–3.88)	3 (2–4.5)	3.5 (2.5–5.63)
Duration of antimicrobial (days)	16 (12–20.5)	16 (11.5–17.25)	17 (14–29)	16 (12.75–23)
Number of drugs mean (Std)	3 (3–4)	3 (3–3.25)	3 (3–4)	3 (3–4)
Mortality n (%)	1 (± 9)	0 (± 0)	0 (± 0)	0 (± 0)

*F* female intact, *FS* Female spayed, *M* Male intact, *MC* Male castrated

^*^Wilcoxon rank sum *P* < 0.05 compared to other two categories

^#^One missing value APPLE_fast_: Acute Patient Physiologic and Laboratory Evaluation score. Std: standard deviation

**Fig. 2:**
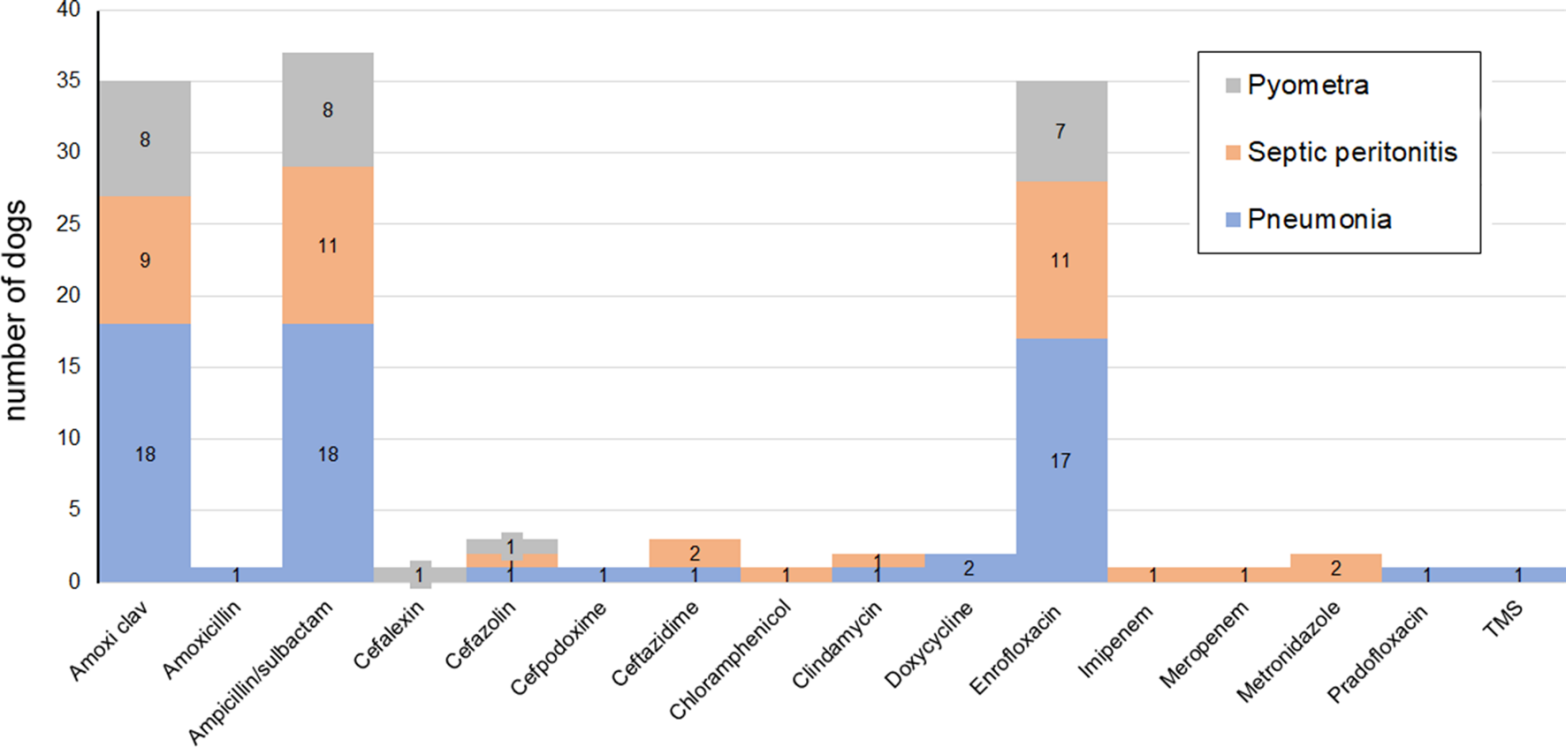
Antimicrobial drugs prescribed by disease process. *Amoxi/Clav* Amoxicillin/clavulanate; *TMS* Trimethoprim-sulfadiazine

Ampicillin/sulbactam, amoxicillin/clavulanate and enrofloxacin were most frequently prescribed (Fig. 2). The median (min–max) number of AMDs prescribed was 3 (2–7) (Table 2) for a median duration of 16 days (9–41). No difference in number of AMDs prescribed or duration of AMD treatment was observed between disease groups (*P* = 0.65 and *P* = 0.20, respectively). Duration of AMD treatment was not correlated with length of hospitalization (ρ 0.02, *P* = 0.89). Dogs received numerous additional medications of which the most common were intravenous fluids (Plasma-Lyte A, LRS, 0.9% NaCl) with electrolyte supplementation (potassium chloride, and/ or dextrose), injectable and oral pain medication (fentanyl n = 15, methadone n = 14, ketamine n = 8, lidocaine n = 4, gabapentin n = 4, non-steroidal-anti-inflammatory drugs n = 2, amantadine n = 1); gastrointestinal medications (maropitant citrate n = 16, metoclopramide n = 12, proton pump inhibitor n = 26, appetite stimulant n = 6, fenbendazole n = 3), vasopressors (norepinephrine n = 5, dobutamine n = 2, phenylephrine n = 1), liver support (acetylcysteine n = 2, S-adenosyl methionine n = 2, phytonadione n = 1), anticonvulsants (zonisamide n = 2, phenobarbital n = 1, levetiracetam n = 1), cardiac medication (sildenafil n = 2, furosemide n = 1), immunomodulators (azathioprine n = 2, glucocorticoids n = 1), albumin transfusion (n = 3), topical ocular drugs n = 2, insulin, pyridostigmine, rivaroxaban and aminocaproic acid all n = 1. Oxygen supplementation was administered to 7 dogs. Diet was not controlled during the study and dogs were fed a variety of dry and wet commercial canine diets, as well as homemade diets throughout the study period. During hospitalization 11 dogs received enteral nutrition via nasogastric feeding tube (Clinicare™, Abbott laboratories, CA, USA). Probiotics (Fortiflora®, Purina Pro Plan Veterinary Supplements®, Nestle SA, Switzerland) were prescribed to 2 dogs during and after hospitalization.

**Table 2:** Number of antimicrobial drugs per dog based on disease types prescribed during the study duration

Number of antimicrobial drugs	Septic peritonitis (n = 11)	Pyometra (n = 8)	Bacterial pneumonia (n = 17)	All dogs (n = 36)
2	1	1	0	2
3	6	5	11	22
4	2	2	5	9
5	1	0	0	1
6	0	0	1	1
7	1	0	0	1

### Microbiological analyses

**Fig. 3:**
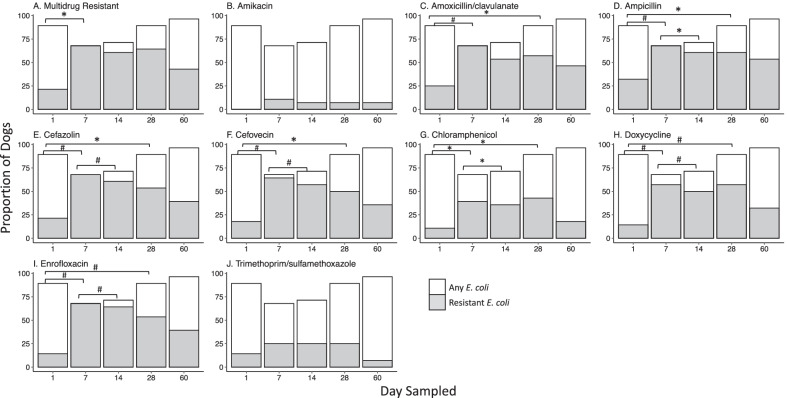
Fecal *E. coli* susceptibility over time. Percent of MDR-*E. coli* at different time points are shown in panel A, with total percentage of dogs with any *E. coli* cultured in white, and MDR-*E. coli* shaded grey. Panels B through J depict susceptibility for the individual antimicrobial drugs tested. In those images, the white boxes also indicate the percentage of dogs with any *E. coli*, and the shaded boxes indicate the proportion of dogs with resistance to each respective drug as labeled. Statistically significant differences (Exact McNemar test) are denoted by symbols *P ≤ 0.05 and #*P* ≤ 0.01

**Fig. 4:**
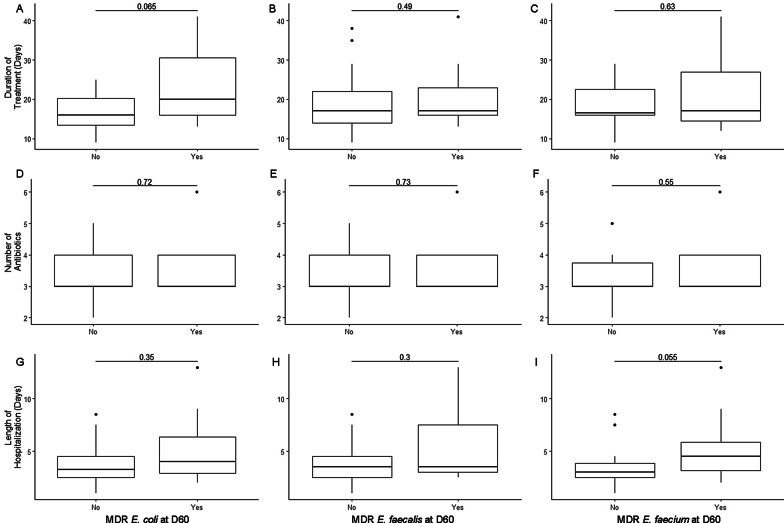
Whisker plot showing the lack of association of duration of antibiotic treatment, number of antibiotics administered, and length of hospitalization in relation to dogs with and without a MDR *E. coli*, *E. faecalis*, or *E. faecium* at D60 with Wilcoxon rank sum test *P*-values

**Fig. 5:**
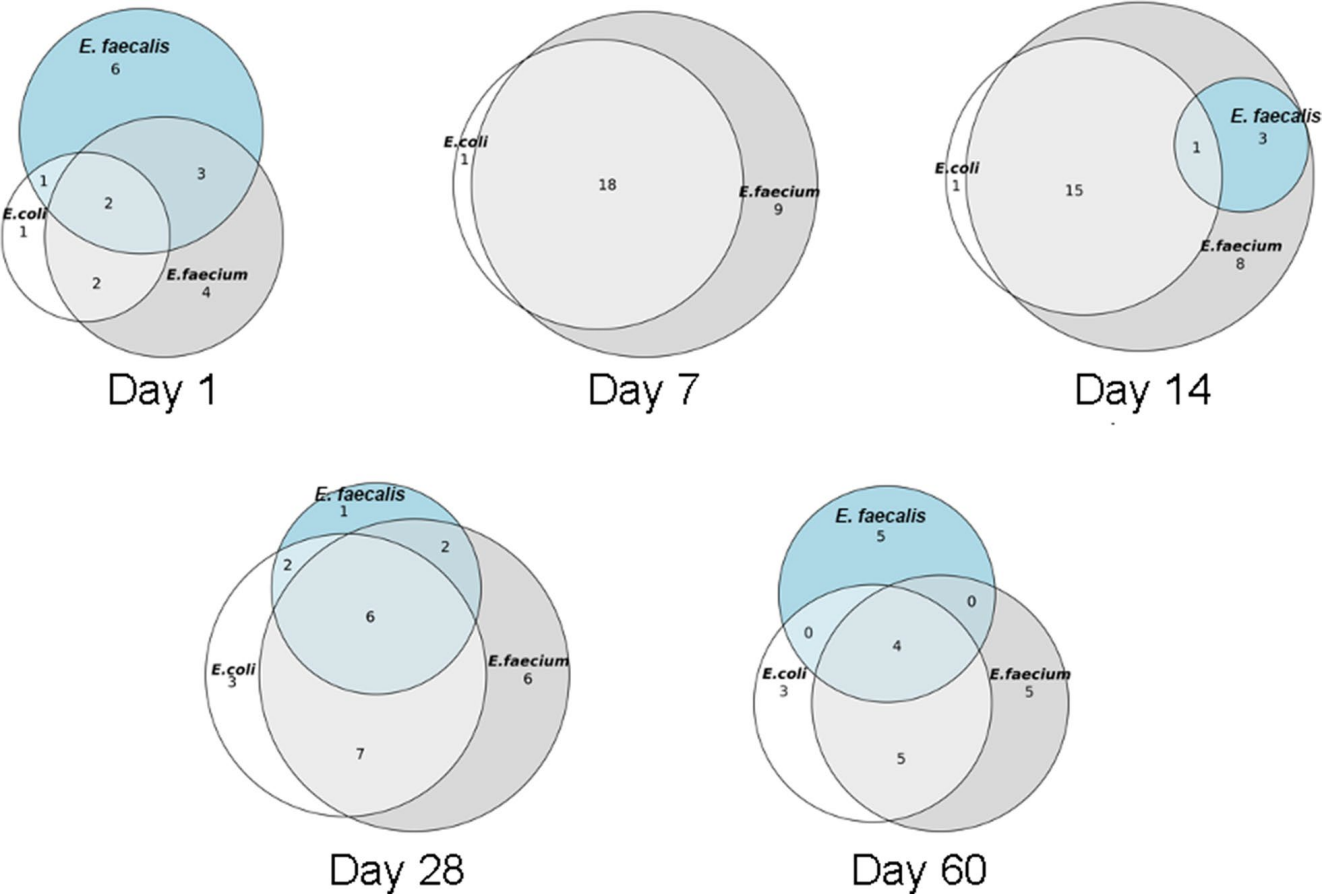
Venn diagrams displaying distribution of dogs in which a MDR pathogen was recovered from fecal samples at the different time points. For example, on day 1, one dog had 1 MDR *E.coli* only isolated, 6 dogs had MDR *E. faecalis* only isolated, 4 dogs had MDR *E. faecium* only, one dog had MDR *E.coli* and *E. faecalis* recovered, 2 had MDR *E.coli* and *E. faecium*, 3 had MDR *E. faecalis* and *E. faecium* recovered, and 2 dogs had all 3 MDR pathogens isolated

Microbiological analysis, specifically isolation of *E.coli* and *E. faecalis* and *E*. *faecium* and respective AMD susceptibility testing was performed to determine changes during and after AMD exposure. Microbiological analysis was performed for the 28 dogs for which a sample was obtained at each of the 5 study time points. *E. coli* was recovered from 25/28 (89.3%) dogs on D1, from 19/28 (67.9%) on D7 (D1 vs D7, *P* = 0.07) and from 27/28 (96.4%) samples on D60 (D7 vs. D60, *P* = 0.008). The frequency of recovering an MDR *E. coli* increased from 21.4% of dogs on D1 to 67.9% of dogs on D7 (D1 vs D7, *P* < 0.001) and decreased to 42.9% of dogs by D60 (D7 vs D60 *P* = 0.04) (Fig. 3). This pattern of increasing resistance between D1 and D7 with subsequent decreases by D60 was common to most AMDs evaluated (Fig. 3). All *E. faecalis* and *E. faecium* isolates recovered were MDR at all time points. The frequency of *E. faecalis* isolation was lower at D7 compared to D1 and D60 (*P* < 0.001 and *P* = 0.004, respectively), with no difference between frequency at D1 compared to D60 (*P* = 0.61) (Additional file 1: Figure S2). In contrast, the frequency of *E. faecium* was greater at D7 compared to D1 and D60 (both *P* < 0.001). The frequency of *E. faecium* was not different between D1 and D60 (*P* = 0.55) (Additional file 1 Figure S2). *E. faecium* isolates on D1 were resistant to numerous AMDs including amikacin, cefazolin, clindamycin, enrofloxacin, erythromycin, trimethoprim-sulfamethoxazole, oxacillin (39.3% of dogs with a resistant *E. faecium* each), nitrofurantoin (35.7%), rifampin (32.1%), penicillin, doxycycline, and imipenem (28.6% each) (Additional file 1: Figure S3). Duration of AMD treatment, number of AMDs administered, and length of hospitalization were not associated with recovery of MDR *E. coli*, *E. faecalis*, or *E. faecium* at D60 (Fig. 4). The distribution of MDR in *E. coli*, *E. faecalis* and *E. faecium* isolates over time is displayed in Fig. 5.

### Antimicrobial resistance genes

**Fig. 6:**
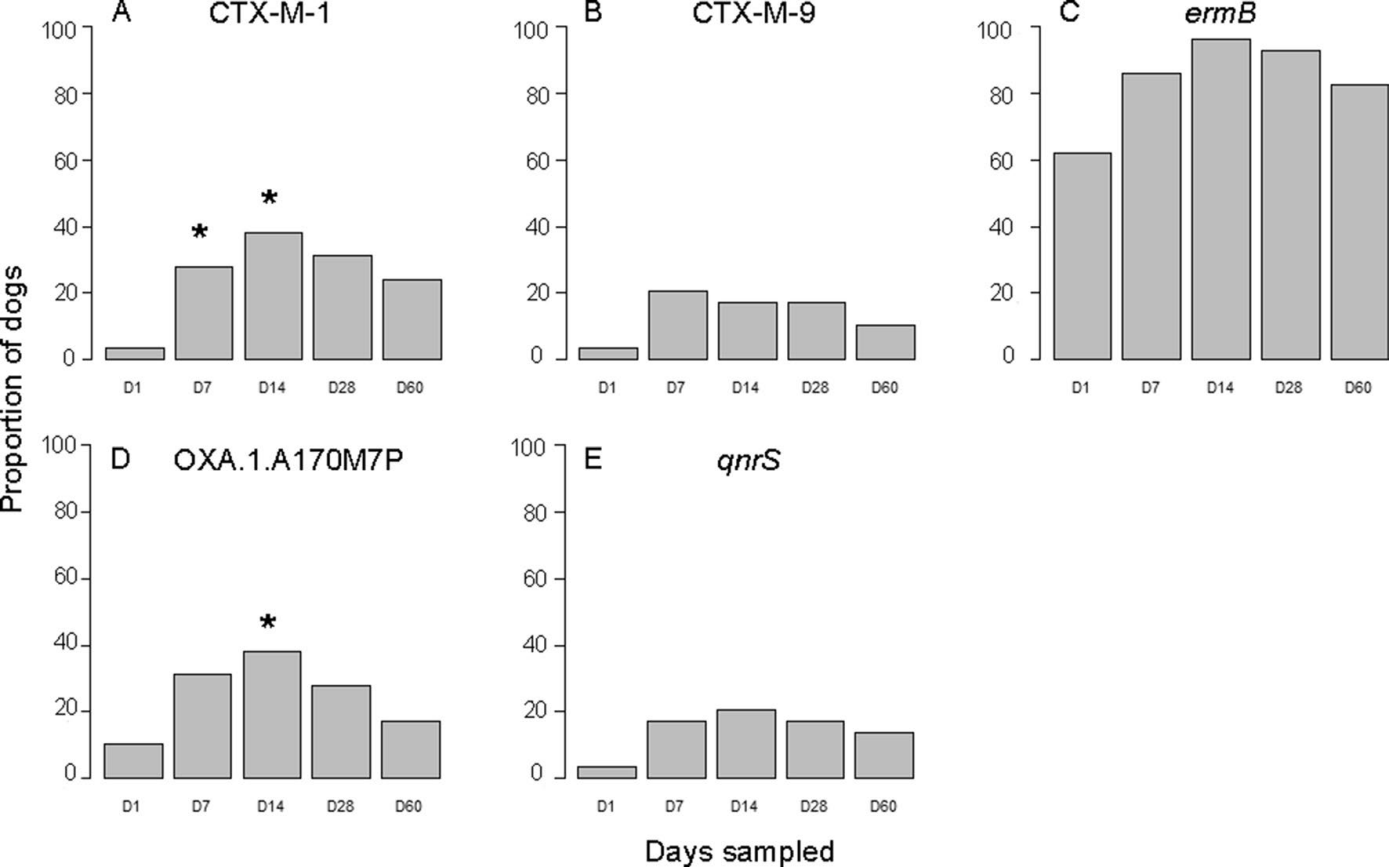
Bar histogram showing the proportion of dogs in which a resistant gene was recovered compared to the all the dogs at the different time points. Frequency of recovery of resistance gene was compared between peak time point and D1 using the McNemar test. *P ≤ 0.05

**Fig. 7:**
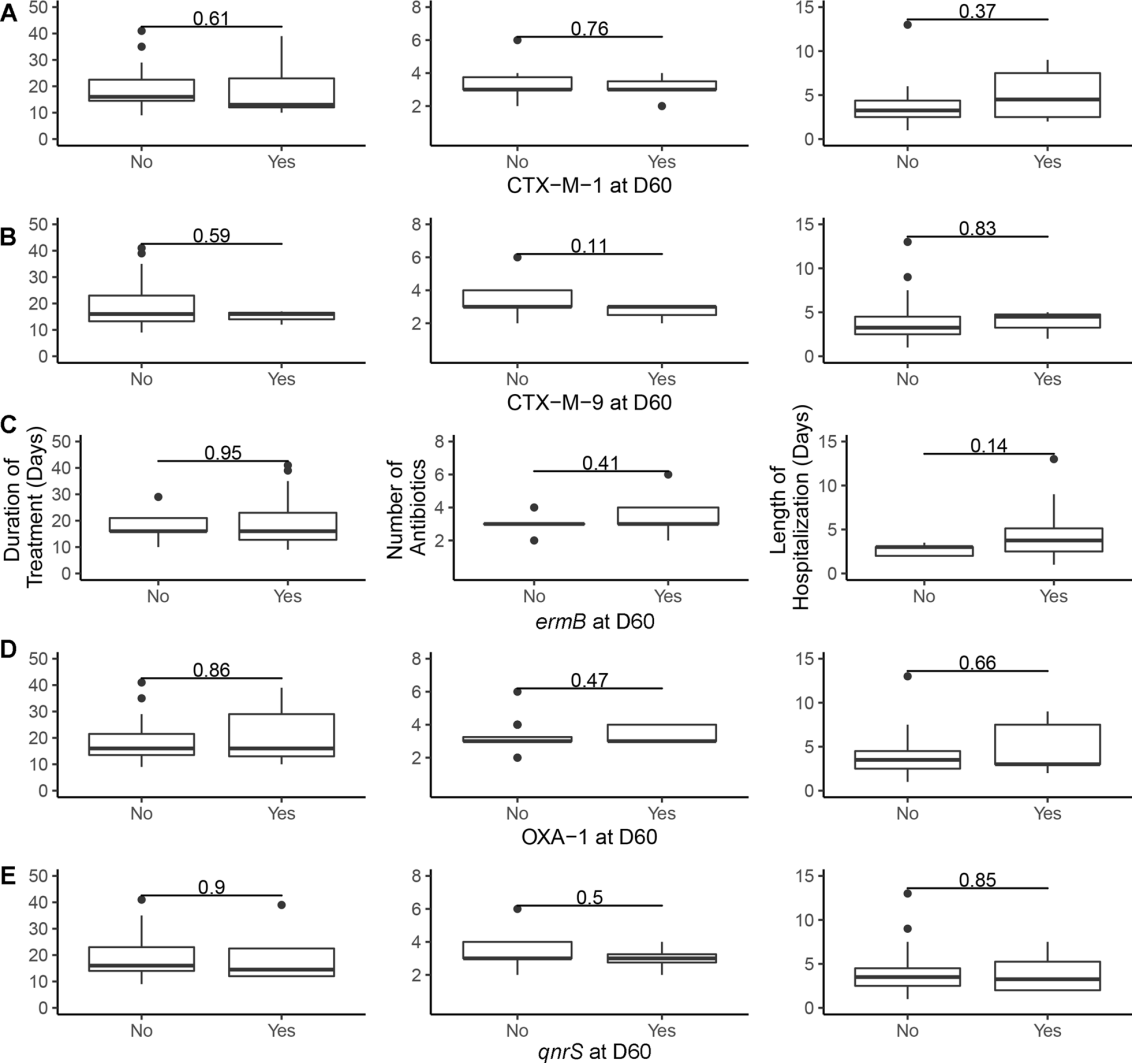
Whisker plot showing the lack of association of duration of antibiotic treatment (left panels), number of antibiotics administered (middle panel), and length of hospitalization (far right panel) and recovery of AMR genes at Day 60 (panels A to D for CTX-M-1; CTX-M-9; *erm*B, OXA-1 and *qnr*S respectively) with Wilcoxon rank sum *P*-values

Presence or absence of 17 AMR genes corresponding to the drugs used for microbiologic susceptibility testing was performed over time to describe the fecal resistome and ascribe presence of AMR genes to phenotypic AMR via microbiological testing. DNA extraction was performed on 145 fecal samples from 29 dogs from all 5 time points. Of the 17 genes targeted, only 5 AMR gene targets were detected in ≥ 2 samples: *ermB* (n = 122), CTX-M-1 (n = 36), OXA-1 (n = 36), *qnrS* (n = 21), and CTX-M-9 (n = 20). All reached maximum prevalence on D14 and decreased by D60, except CTX-M-9, which peaked on D7 (Fig. 6). Detection of *ermB*, CTX-M-1, and OXA-1 was significantly more frequent at the corresponding peak time point than on D1 (*P* = 0.002, *P* = 0.002, and *P* = 0.039, respectively). There were no differences in frequency of *qnrS* and CTX-M-9 between time points. There was no association between AMD treatment duration, number of AMDs administered, or length of hospitalization and AMR gene prevalence at D60 (Fig. 7).

Phenotypic AMR data from *E. coli*, *E. faecium*, and *E. faecalis* was available for 24/29 dogs with AMR gene detection data. Detection of *ermB* was associated with recovery of a clindamycin- and erythromycin-resistant *E. faecium* isolate (Odds Ratio (OR) 4.0, (95% Confidence Interval (CI) 1.3–13.2), *P* = 0.009) but not recovery of a resistant *E. faecalis* isolate. Detection of CTX-M-1 was associated with isolation of a cefpodoxime-resistant *E. coli* (OR 12.1, 3.3–68.0, *P* < 0.001) but not a cefpodoxime-resistant *E. faecium* or *E. faecalis*. Detection of OXA-1 was associated with isolation of *E. coli* resistant to ampicillin (OR 5.1, 1.6–22.3, *P* = 0.003), amoxicillin/clavulanate (OR 6.1, 1.8–26.3, *P* = 0.001), and cefpodoxime (OR 3.8, 1.4–12.0, *P* = 0.006). There were no associations between OXA-1 detection and ampicillin, amoxicillin/clavulanate, cefpodoxime, or oxacillin resistance in *E. faecium* or cefpodoxime or oxacillin resistance in *E. faecalis*. Detection of *qnrS* was not associated with isolation of an enrofloxacin-resistant *E. coli*, *E. faecium*, or *E. faecalis*. No significant associations were found between CTX-M-9 detection and isolation of a cefpodoxime-resistant isolate of any organism. Associations with ampicillin and amoxicillin/clavulanate were not tested in *E. faecalis* because of low numbers of resistant isolates (1 and 0, respectively).

### Microbial composition

**Fig. 8:**
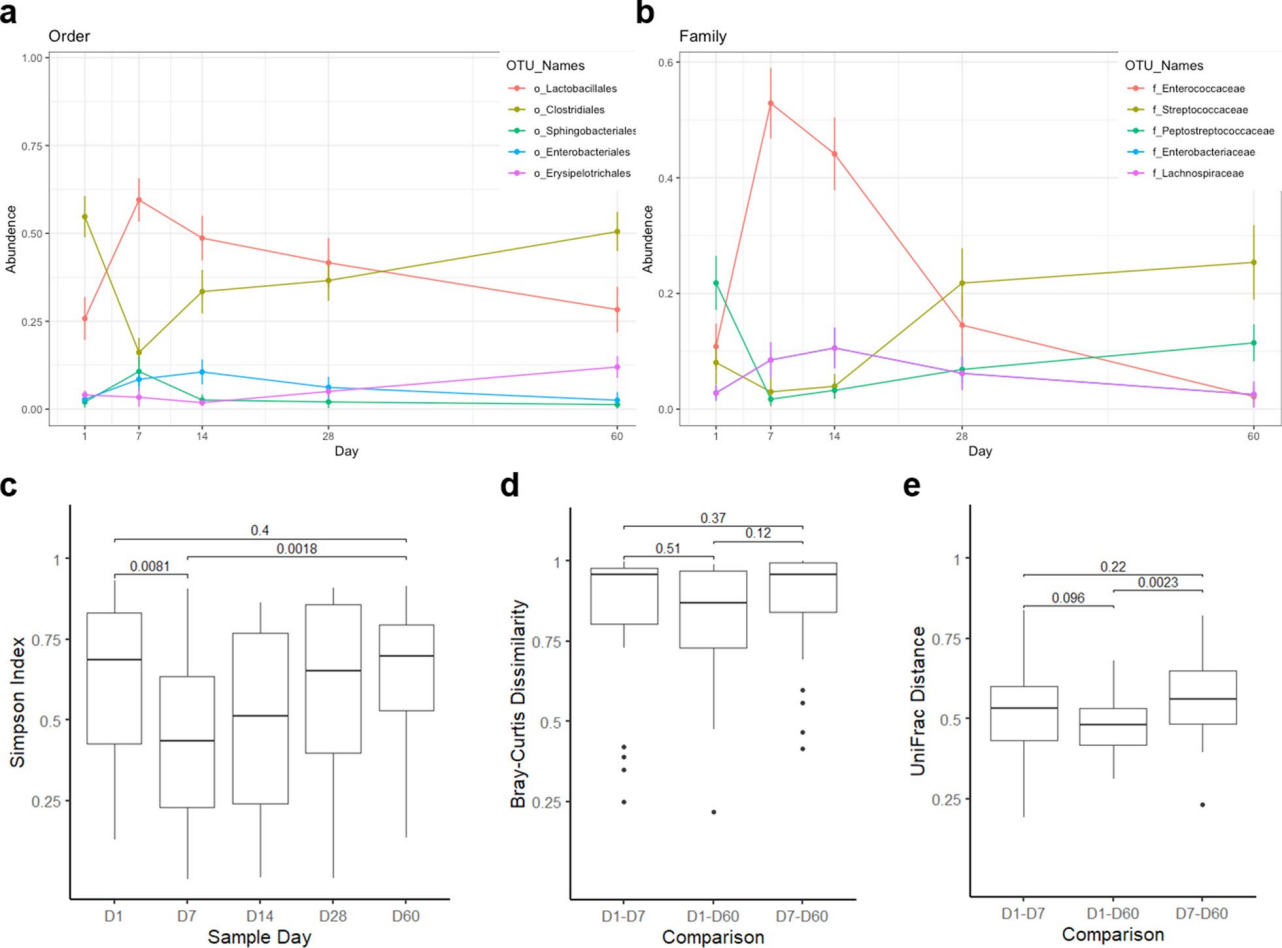
Changes in fecal microbiota composition over the study period. Taxa are visualized by order (**a**) and family (**b**). Points bars represent the median relative abundance and interquartile range of the five most abundant taxa across the 25 samples for which 16S data were available at all 5 time points. Families are given the same color as their parent orders. Simpson’s Diversity Index of samples at each time point, with Wilcoxon signed rank *P*-values comparing D1, D7, and D60 samples (**c**). Beta diversity between D1, D7 and D60 samples from the same patient, measured by Bray–Curtis dissimilarity (**d**) and UniFrac distance (**e**) and labeled with Wilcoxon signed rank *P*-values

**Fig. 9:**
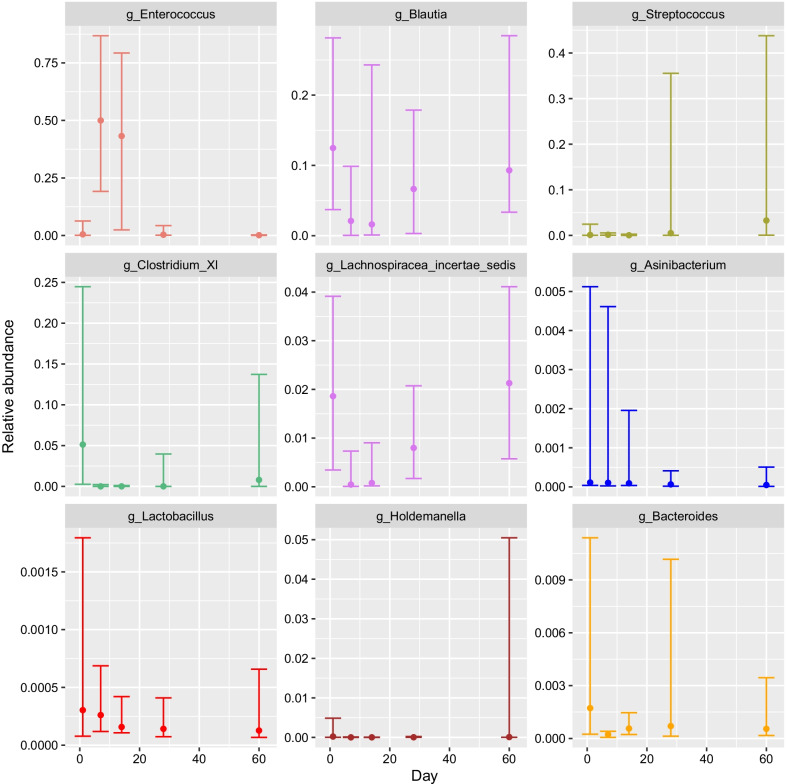
Changes in relative abundance of nine most abundant bacterial genera over time. Points bars represent the median relative abundance and interquartile range

The fecal microbiome was analyzed during and following AMD administration to determine the impact of AMD on the microbiome and its recovery following AMD discontinuation. A total of 25 dogs had ≥ 10,000 16S rRNA reads at each of the 5 time points and were included in analyses of microbial composition. Alpha diversity, as measured by Simpson’s diversity index, decreased from D1 to D7 (*P* = 0.008) and increased across the remaining time points (Fig. 8C). Alpha diversity was decreased on D7 compared to that at D60 (*P* = 0.002), but there was no difference in alpha diversity between D1 and D60 (*P* = 0.4). There was no correlation between alpha diversity on D60 and length of hospitalization, duration of AMD treatment or number of AMDs administered. Beta diversity measures also suggested a pattern of initial disruption followed by increases over time. The median Bray–Curtis dissimilarity between D1 and D60 samples from the same animal was lower than that between D1 and D7 and D7 and D60, but these differences were not significant (Fig. 8D). Comparisons of UniFrac distance showed a similar pattern, and the distance between D7 and D60 samples was larger than that between D1 and D60 samples (*P* = 0.002) (Fig. 8E). Changes in phylum and class over time are shown in Additional file 1: Figure S4. The primary orders present were Clostridiales and Lactobacillales. Clostridiales predominated at D1 with a median relative abundance of 60.0% (IQR 19.9–83.6%) but declined to 4.6% (0.1–20.4%) by D7 (*P* < 0.001). The abundance of Clostridiales increased over subsequent time points (D14, D28) and on D60 (59.7%, IQR 34.3–83.6%) was not different from D1. The two most common families within the Clostridiales, Lachnospiraceae and Peptostreptococcaceae, both echoed this pattern. The Lactobacillales followed the opposite trajectory, with relative abundance increasing from 3.8% (0.3–62.6%) on D1 to 61.7% (27.1–91.5%) on D7 (*P* = 0.005) and then decreasing gradually from D14 and D28 to 4.8% (0.5–33.4%) on D60 (Fig. 8A). On the family level, this pattern was most apparent within the Enterococcaceae, although the D60 abundance (0.1%, 0.0–0.2%) was lower than that observed at D1 (0.9%, 0.1–2.6%) (*P* = 0.02) (Fig. 8B). The only difference between time points for the next most abundant Lactobacillales family, the Streptococcaceae, was between D7 (0.2%, 0.1–0.4%) and D60 (3.0%, 0.0–13.9%) (*P* = 0.002). Among the next three most abundant orders, the Erysipelotricacales increased from a D1 abundance of 0.5% (0.4–2.2%) to a D60 abundance of 2.6% (1.0–14.2%) (*P* = 0.02), the Enterobacteriales increased from D1 (0.1%, 0.0–0.5%) to D7 (1.6%, 0.0–4.9%) (*P* = 0.03) and returned to baseline by D60 (0.1%, 0.0–0.7%), and the Sphingobacteriales increased from D1 (0.0%, 0.0–0.2%) to D7 (0.0%, 0.0–0.6%) (*P* = 0.03) and returned to baseline by D60 (0.0%, 0.0–0.1%). Changes within the most abundant bacterial genera are shown on Fig. 9. *Enteroccocus* increased from D1 (0.0%, 0.0–0.0%) to D7 (0.5%,0.2–0.9%) and returned to baseline by D60 (0.0%, 0.0–0.0%). A similar pattern was seen with *Asinibacterium*. *Holdemanella* increased from D1 to D7, followed by a return to baseline and sharp increase between D14 and D60. *Blautia*, *Clostridium XI*, *Lachnospiracea incertae sedis,* all followed a similar pattern, with decrease in abundance in D7 compared to D1, followed by a return to baseline by D60. *Lactobacillus* and *Bacteroides* decreased between D1 and D7, followed by a return to gradual return to baseline by D28, and decreased compared to peak abundance by D60. Streptococcus increased on D28 (0.0%, 0.0–0.4%) and D60 (0.0%, 0.0–0.4%) compared to D1 (0.0%, 0.0–0.0%).

## Discussion

This study documents the effects of AMDs on the fecal microbiota of critically ill dogs during and after hospitalization. At hospital admission, dogs with sepsis in the present study had similar microbiomes to those of healthy dogs, which are characterized by highly prevalent Fusobacteria, Firmicutes and Bacteroidetes, with Actinobacteria, Proteobacteria, Cyanobacteria and Verrucomicrobia (which includes enterococcal, clostridial, bifidobacterial and eubacterial groups) in the minority [22–26]. At baseline, the microbiota of dogs with sepsis were predominantly Firmicutes and Bacteroidetes, followed by Proteobacteria, Actinobacteria, and Fusobacteria. Consistent with a prior study of dogs in an intensive care unit receiving AMDs, dogs in the present study had significant increases in Enterococcaceae above the normal low prevalence in the microbiome of healthy dogs [27]. In the present study, AMD treatment caused significant decreases in taxonomic richness and diversity consistent with other populations [28–31], and produced changes in microbiota after AMD discontinuation with incomplete return to baseline taxonomic profiles at the family level after 2 months, as described in humans [32].

Our study assessed the impact of AMD treatment on fecal *E. coli*, a commensal in the gastrointestinal tract of dogs, which is a known cause of sepsis [33–35]. Drug resistance in pathogenic *E. coli* in dogs is concerning, with a hospital-associated cluster of carbapenem resistance reported in 2018 in the US [36]. In our study, AMD administration was associated with selection of numerous instances of resistance to different AMD classes, including those to which the dogs were not exposed. These results confirm previous findings, where treatment with amoxicillin/clavulanate is associated with development of resistance to third-generation cephalosporins in *E. coli* [9, 37]. Similarly, treatment with a fluoroquinolone is associated with development of fluoroquinolone-resistant and cephalosporin-resistant *E. coli* [10, 38]. Those effects were transient for most AMDs and subsided by D28, consistent with recovery of susceptibility to amoxicillin/clavulanate in *E. coli* between 2 and 3 weeks after AMD discontinuation in healthy dogs and those treated for acute diarrhea [9, 38, 39]. The median duration of treatment in the present study was 16 days, suggesting that return of susceptibility occurs within 10 days of discontinuation even in critically ill dogs. Dogs with gastrointestinal disease might have additional alterations in the gastrointestinal microbiota that might prolong recovery after AMD administration [39].

AMD administration was associated with selection of MDR *E. coli* in hospitalized dogs, but duration of hospitalization was not, unlike in previous studies [11, 40]. The lack of association between hospitalization duration and MDR frequency might relate to stringent biosafety measures limiting environmental contamination in our hospital. Other documented risk factors associated with the development of MDR *E. coli* during hospitalization include administration of cephalosporins, and/ or metronidazole while hospitalized [40]. We did not specifically investigate drug association with MDR *E. coli* status because > 90% of dogs were prescribed at least amoxicillin/clavulanate and a fluoroquinolone. In addition, unlike a previous veterinary study [37], we found no association between AMD treatment duration and the frequency of MDR *E. coli* at D60. Differences in populations preclude direct comparison between studies, but dogs in the present study received broad spectrum AMD treatment for a prolonged duration. It is noteworthy that AMD treatment duration in the present study was longer than current human medical guidelines for similar diseases. For instance, AMD treatment duration for intra-abdominal infection in humans is typically 5–7 days [41], while for ventilator-associated pneumonia it is commonly 7–8 days [42].

Enterococci are commensal bacteria in the gastrointestinal tract of dogs where they play fundamental roles in food digestion and metabolism. Nevertheless, *Enterococcus* spp. are associated with severe nosocomial infections in humans and animals [43–45], potentially because of extensive AMD resistance, expression of virulence traits including biofilm generation and capacity for horizontal gene transfer [46]. Similar to studies from Europe and the US [44, 47–49], more than 25% of dogs on D1 carried resistant *E. faecium* isolates to numerous AMDs including amikacin, cefazolin, clindamycin, enrofloxacin, erythromycin, trimethoprim-sulfamethoxazole, oxacillin, nitrofurantoin, rifampin, penicillin, doxycycline, and imipenem. Despite this high AMR prevalence, we identified further increases in the number of dogs with resistant *E. faecium* between D1 and D7 to ampicillin, amoxicillin/clavulanate, doxycycline, imipenem, minocycline, nitrofurantoin, penicillin, rifampin, and tetracycline. The documented carbapenem resistance despite very limited usage (1/36 dogs) is particularly concerning because the WHO classifies carbapenems as highest priority agents on the list of critically important AMDs for human health. The selection of AMR for ampicillin, doxycycline, and nitrofurantoin in the present study is similar to another ICU dog population [27], albeit with lower aminoglycoside resistance, perhaps related to the limited usage in the present study [27, 50]. Previous studies reported high prevalence of erythromycin resistance in *E. faecium* [27, 47, 49, 50], consistent with that seen here.

The overall prevalence of AMR in *E. faecalis* was high at admission and was maintained throughout the study period; consistent with data suggesting *E. faecalis* harbor high levels of AMR for chloramphenicol and gentamycin and MDR for up to 9 drugs [44, 47–49]. Given the high prevalence of MDR *Enterococcus* spp., dogs might act as reservoirs of resistant pathogens capable of zoonotic spread. Surprisingly in the present study, no *E. faecalis* isolates were recovered on D7. This could be secondary to increased sensitivity of *E. faecalis* to beta-lactams than *E. faecium* [51], failures in microbiologic isolation or might reflect shifts within the Enterococcaceae population under AMD selection pressure. Moreover, our molecular microbiota data showed a significant increase in abundance of Enterococcus on D7, which argues our findings reflect shifts within Enterococcaceae strains under AMD selection pressure.

Bacteria developed AMR secondary to the AMD treatment by expressing AMR genes that were likely already present in the gastrointestinal resistome. These genes may have been harbored by commensal organisms that we did not culture. To detect these genes in a species-independent manner, fecal samples were directly tested using a commercial diagnostic PCR panel. In the present study, AMD treatment selected for extended spectrum beta-lactamase (ESBL) genes, providing resistance to broad spectrum cephalosporins and penicillin. Consistent with prior studies, AMD administration led to selection of Group 2 serine beta-lactamase genes, specifically subgroup 2be (CTX-M1) and 2d (the penicillinase OXA-1). In a recent study of Enterobacteriaceae isolated from dogs in a German veterinary teaching hospital, 59.7% of isolates carried OXA-1, 16.4% CTX-M-1 and 59.7% of isolates carried CTX-M15 [52]. As expected, there was a positive association between isolation of ESBL genes and phenotypic resistance to first- and third-generation cephalosporins in the present study, consistent with other companion animal *E. coli* isolates [53], where resistance to ampicillin, amoxicillin/clavulanate, cefotaxime, cefpodoxime, ceftazidime and tetracycline was prevalent. Third-generation cephalosporins are frequently prescribed in veterinary medicine [54, 55] and their use has been increasing, potentially due to availability of veterinary approved drugs and their ease of administration. In our population, only 4/37 dogs were prescribed a third-generation cephalosporin, but use of amoxicillin and fluoroquinolones have been associated with selection of resistance to third-generation cephalosporins elsewhere [37].

In the present study, phenotypic fluoroquinolone resistance arose within 7 days of treatment and persisted for a month. Interestingly, this was not associated with increase in isolation of fluoroquinolone plasmid- mediated quinolone resistance (PMQR) genes (*qnrA* and *qnrS*). This is similar to other dog populations where PMQR prevalence was low with *aaac(6’)-Ib-cr* being most prevalent [53, 56], suggesting that PMQR are of limited importance to fluoroquinolone resistance in *E. coli* [57]. Lincosamide and macrolide resistance genes were more frequently recovered on D14 and D28, compared to D1 but did not translate into decreased AMD susceptibility in *Enterococcus*. Macrolide resistance is common in fecal *Enterococcus* from dogs [48, 49] and has been identified in both healthy animals [58] and dogs presented to veterinary practices [48]. Our study confirms that *Enteroccocus* can serve as a reservoir for macrolide and lincosamide resistance, with selection of resistance genes within a month of the start of AMD treatment. Given that a lincosamide (clindamycin) was prescribed in only 2/36 dogs, co-selection of resistance genes is likely. Dogs in the present study received largely consistent AMD dosages, particularly for intravenous formulations. It is not known if dose variation might have impacted the results of the present study, particularly with regards to the frequency of isolation of MDR bacteria. Future studies might be directed at evaluating the relationship between AMD dosage and MDR development.

Our study had some limitations. In many cases, we did not obtain sufficient fecal sample to perform both microbiological analysis and DNA extraction for AMR gene isolation and microbiome analysis. Many dogs were anorexic or had diarrhea limiting sample availability on D1/D7, while only small fecal samples were provided during recheck study visits (D7, D14, D28, D60). In addition, dogs were treated with numerous drugs in addition to AMDs during their hospital stay and recovery. These drugs may have influenced the composition of the fecal microbiome. Lastly, diet was not controlled in this observational study of critically ill dogs, and dogs were fed a wide range of commercial and homemade diets that might also have influenced the composition of the microbiome at the different time points.

## Conclusions

Overall, the present study suggests that AMD drugs exert substantial selection pressure on the gastrointestinal microbiota with potential long-term effects, as suggested by previous studies [59–61]. The prevalence of MDR isolates in dogs receiving AMDs and the long duration of AMD treatment documented in the present study highlights the need for strategies to improve AMD stewardship and safely reduce the duration of AMD treatment in veterinary medicine.

## Methods

### Study population

Dogs treated at the Cornell University Hospital for Animals from 06/2017–06/2019 for septic peritonitis, pyometra or bacterial pneumonia were eligible for study if they had received AMDs for ≤ 24 h prior to enrollment. Septic peritonitis was diagnosed by a positive bacteria culture of peritoneal effusion, identification of intracellular bacteria on peritoneal fluid cytology, and/or visualized perforation of the gastrointestinal tract or evidence of free gas within the peritoneal cavity unrelated to abdominal surgery within the past 30 days [62]. Pyometra was diagnosed in intact female dogs with compatible history and clinical signs, diagnostic imaging consistent with a distended fluid filled uterus and confirmed following surgical excision of the uterus [63]. Bacterial pneumonia was diagnosed in dogs with acute onset respiratory distress, cough, tachypnea (respiratory rate > 30 bpm) or hyperventilation (PaCO_2_ < 35 mmHg), a recent known risk factor for aspiration pneumonia (anesthesia or sedation, regurgitation or vomiting, laryngeal or pharyngeal dysfunction, esophageal or neurologic disease) or community-acquired pneumonia (communal housing, exposure to a contagious respiratory pathogen, prior upper respiratory tract disease) and consistent radiographic abnormalities [64]. This study was approved by the Cornell University Institutional Animal Care and Use Committee (Protocol #2014-0053), and dogs were enrolled with written, informed client consent. Dogs that were lost to follow-up, or that died or were euthanized prior to obtaining a second study sample (day 7) were excluded (Additional file 1: Fig. S1). Respective primary clinicians determined all aspects of clinical patient management, including the type and duration of AMDs administered. For each dog, age, breed, sex, bodyweight, illness severity score [65], final diagnosis, type and duration of AMDs administered, length of hospitalization, and outcome were recorded.

### Fecal sample collection

At least 1 g of feces were collected from passed stool or by gentle digital sampling with a sterile glove at enrollment (D1), day 7 (D7), day 14 (D14), day 28 (D28) and day 60 (D60). Feces were divided into 2 aliquots; one sample was submitted for microbiological fecal analysis after refrigeration for a maximum of 36 h if required; the other was stored frozen at − 80 °C pending batch processing.

### Microbiological fecal analysis

For *E. coli* detection, fresh fecal samples were plated directly onto blood agar and EMB plates and incubated overnight at 35 °C with 5% CO_2_. Two probable *E. coli* colonies were manually picked from the EMB plate and bacterial identification confirmed by matrix-assisted laser desorption ionization-time of flight mass spectrometry (MALDI-TOF) (Bruker Microflex LT/SH with Real Time Classification v3.1 and reference library 7854). Automated AMD susceptibility testing using a range of AMDs (Fig. 1A) was performed after 18 h of incubation by broth microdilution per CLSI guidelines [66], using commercial Gram-negative plates (Sensititre, Companion Animal Gram Negative, COMPGN1F Vet AST plate, Thermo Fisher Scientific, Waltham, MA). Minimum Inhibitory Concentration and interpretations (Sensitive, Intermediate, Resistant) were made using proprietary software (Sensititre SWIN Software System, Thermo Fisher Scientific) with recent CLSI (Vet01-S2, 2013/Vet 08, 2018) breakpoints. For detection of *Enteroccus faecium* and *E. faecalis,* fecal samples were plated onto Trypticase soy agar with 5% sheep blood (Becton Dickenson GmBh, Heildelberg, Germany) and Columbia CNA plates (Becton Dickenson GmBh, Heildelberg, Germany) and incubated overnight at 35 °C with 5% CO_2_. Six probable *Enterococcus* colonies were manually picked from CNA plates and bacterial ID confirmed by MALDI-TOF to obtain at least 2 isolates each of *E. faecium* and *E. faecalis*. Automated AMD susceptibility testing was performed as for *E. coli* using Gram positive isolate plates (Sensititre Companion Animal Gram Positive COMPGP1F Vet AST plate, Thermo Fisher Scientific). Interpretations were as for *E. coli*. To account for multiple isolates per sample, data were collapsed such that any sample with ≥ 1 resistant isolate was classified as resistant. Percentage of dogs carrying resistant isolates was calculated as the sum of intermediate and resistant isolates divided by total isolates. AMD class used to determine multidrug resistant status, defined as resistance to ≥ 1 agent in ≥ 3 AMD categories per Magiorakos and others, 2012 [67]. One biological and technical replicate was performed for each sample.

### DNA extraction

For direct molecular analysis, a 290–310 mg subsample of feces from each dog was homogenized in 800 μL PBS (Mini‐Beadbeater‐96, BioSpec Products, Bartlesville, OK). Homogenates were centrifuged (5 min, 1500 × g, room temperature), and DNA extracted from 175 µL of supernatants using a commercial kit (MagMAX CORE Nucleic Acid Purification Kit, Thermo Fisher Scientific) on an automated extraction instrument (Kingfisher Flex, Thermo Fisher Scientific) with an additional mechanical lysis step with zirconia beads (2 × 2.5 min, 40 oscillations/s with a 5-min rest, Mini‐Beadbeater‐96, BioSpec Products, Bartlesville, OK). Two negative extraction controls (PBS) were included on each extraction plate. The DNA from each sample was split into two aliquots (one for qPCR and one for Microbiota analyses) and frozen at − 80 °C until further processing.

### Quantitative PCR for resistance genes

Nanoliter-scale real-time quantitative PCR was performed using an integrated qPCR instrument (QuantStudio 12 K Flex OpenArray platform, Thermo Fisher Scientific) using a commercial multiplexed panel (ABRx, Diatherix-Eurofins, Hunstville, AL) detecting 17 AMR genes corresponding to the drugs used for microbiologic susceptibility testing (Fig. 1B) and one internal quality control target to control for inhibition. Briefly, a custom panel-specific preamplification primer mix (Thermo Fisher Scientific #4441856) and TaqMan® PreAmp Master Mix (Thermo Fisher Scientific, #4488593) were used. Reaction volume is fixed at 10 µL and was performed on ABI 9700 thermocyclers with a enzyme activation cycle at 95 °C 1o min, followed by amplification (95 °C, 15 s; 60 °C, 4 min for 10 cycles) and then enzyme deactivation (99 °C, 10 min). Preamplified products were diluted using a 1:10 dilution with nuclease-free water. Real time ABRx™ Panel volume was fixed at 5 µL and used TaqMan® OpenArray® Real-Time PCR Master Mix (Thermo Fisher Scientific, #4462164). The ABRx™ Panel real-time PCR setup utilizes TaqMan® OpenArray® Real-Time PCR Master Mix (Thermo Fisher Scientific, #4462164). Reaction volume is fixed at 5 µL. In short, UDG incubation cycle 50 °C, 2 min was followed by enzyme activation at 95 °C, 10 min and then amplification at 95 °C, 15 s followed by 60 °C, 1 min for 40 cycles. The assay was validated using QuantStudio™ 12K Flex Real-Time PCR System with AccuFill. The analyses were performed in triplicate, using the same clinical cut-off values used in human testing. Panel contents are listed in the company’s technical bulletin [68].

### Microbiota analyses

Fecal DNA was submitted to the Weill Cornell Medicine Microbiome Core Laboratory and quantified using the Quant-iT dsDNA High Sensitivity Assay Kit (Thermo Fisher, Waltham, MA) on the GloMax plate reader (Promega, Madison, WI) using a microplate (Greiner Bio-One, Monroe, NC, part 655087). The Earth Microbiome Protocol with primers targeting the V4 region of the 16S SSU rRNA was used for library preparation [69]. Amplicon libraries were washed using AMPure XP magnetic beads (Beckman Coulter, Brea, CA). Library concentrations were quantified using the same method as for the original DNA. Library quality and size verification was performed using the PerkinElmer LabChip GXII instrument with the DNA 1K Reagent Kit (CLS760673). Individual library peak molarities were calculated based on their peak size and concentration. After normalization at 2 nM, libraries were pooled using the same volume across all normalized libraries. Pooled libraries were sequenced on the MiSeq instrument (Illumina, San Diego, CA) at loading concentration of 7.5 pM with 15% PhiX, paired-end 250 using the MiSeq Reagent Kit v2, 500-cycles (MS-102-2003). Demultiplexed raw reads were processed to generate an operational taxonomic unit (OTU) table using USEARCH version 11.0.667 [70]. Specifically, forward, and reverse reads were merged using a maximum of 5 mismatches in the overlap region, a minimum sequence identity in the overlap region of 90 percent, a minimum overlap length of 16 base pairs, and a minimum merged sequence length of 300 base pairs. PhiX contamination was then removed, followed by quality filtering based on FASTQ quality scores, with a maximum expected error number of 1.0. Operational taxonomic unit clustering was performed using the USEARCH UPARSE-OTU algorithm with default settings. Merged (pre-filter) reads were mapped to the OTU sequences to generate the OTU table. Taxonomic classification of OTU representative sequences was performed using an implementation of the SINTAX algorithm via USEARCH [71], using v.16 of the Ribosomal Database Project (RDP) Training Set [72]. Alpha diversity estimation and principal coordinate analysis (PCoA) were performed using the phyloseq R package [73]. One biological and technical replicate was performed for each sample.

### Statistical analyses

Patient age, bodyweight, illness severity score, length of hospitalization, AMD treatment duration, and number of AMDs administered were compared between the three disease groups using the Kruskal–Wallis test with post-hoc Mann Whitney U tests performed for each pair. Spearman rank correlations were calculated between APPLE_fast_ score and both duration of AMD treatment and length of hospitalization. These correlations were computed for the entire patient population and within disease groups. For analyses of microbiological and qPCR data, only dogs for which a sample was collected at each time point were included in the final analyses. Isolates were classified as resistant if the automated interpretation of susceptibility was intermediate or resistant. If the susceptibility of 2 isolates from the same sample differed, the sample was considered resistant if either isolate was interpreted as resistant. Percentage resistance calculations were conducted on a per sample (i.e., per dog), not per isolate basis. *E. coli* and *Enterococcus* spp. were classified multidrug resistant (MDR) if the isolate was resistant to ≥ 1 agent in ≥ 3 AMD categories (Fig. 1A) [67]. Differences between time points in the frequency of recovery of any isolates and of an MDR isolate were assessed using the McNemar test. The Mann Whitney U test was used to assess whether AMD treatment duration, number of AMDs administered, or length of hospitalization were associated with recovery of MDR isolates at D60. Alpha diversity measured using Simpson’s diversity index, and beta diversity measured using both Bray–Curtis dissimilarity and UniFrac distance were used to assess changes in microbial composition over time. The Wilcoxon signed-rank test was used to compare alpha diversity between D1, D7, and D60 and the beta diversity between each pairwise combination of those three time points. Spearman’s rank correlations were used to assess association between alpha diversity at D60 and length of hospitalization, number of AMDs administered and AMD treatment duration. For any AMR genes detected in > 1 sample but not in all samples, the frequency of detection was compared between D1, D60, and the peak time point using the McNemar test. Associations between gene detections and AMD treatment duration, number of AMDs administered, and length of hospitalization were assessed using the Mann Whitney U test. Fisher’s exact test was used to assess associations between detection of a given AMR gene and isolation of an *E. coli*, *E. faecium*, or *E. faecalis* with corresponding phenotypic AMD resistance. Changes in the frequency of the most common orders and families between time points were assessed using the Wilcoxon signed-rank test. Statistical analyses were performed using R v3.4.2 or v4.0.5 [74].

## Supplementary Information

**Additional File 1. Figure S1.** Patient enrollment summary. **Figure S2.** Fecal Enterococcus faecalis susceptibility over time. **Figure S3.** Fecal Enterococcus faecium susceptibility over time. **Figure S4.** Changes in fecal microbiota composition over the study period.

## Data Availability

Sequence files and metadata for the microbiome analyses have been deposited in NCBI SRA under BioProject PRJNA756887.

## Abbreviations

AMR: Antimicrobial resistance
AMD: Antimicrobial drug
MDR: Multidrug resistance
OUT: Operational taxonomic unit
PCR: Polymerase chain reaction
