# Supplementary material for: Effect of antimicrobial administration on fecal microbiota of critically ill dogs: dynamics of antimicrobial resistance over time
Source: Anim Microbiome. 2022 Jun 4;4:36. doi: 10.1186/s42523-022-00178-9 (PMC9167539; doi:10.1186/s42523-022-00178-9)

Supplemental Materials

Menard et al. "Effect of antimicrobial administration on fecal flora of critically ill dogs: Dynamics of antimicrobial resistance over time."

Figure S1: Patient enrollment summary.

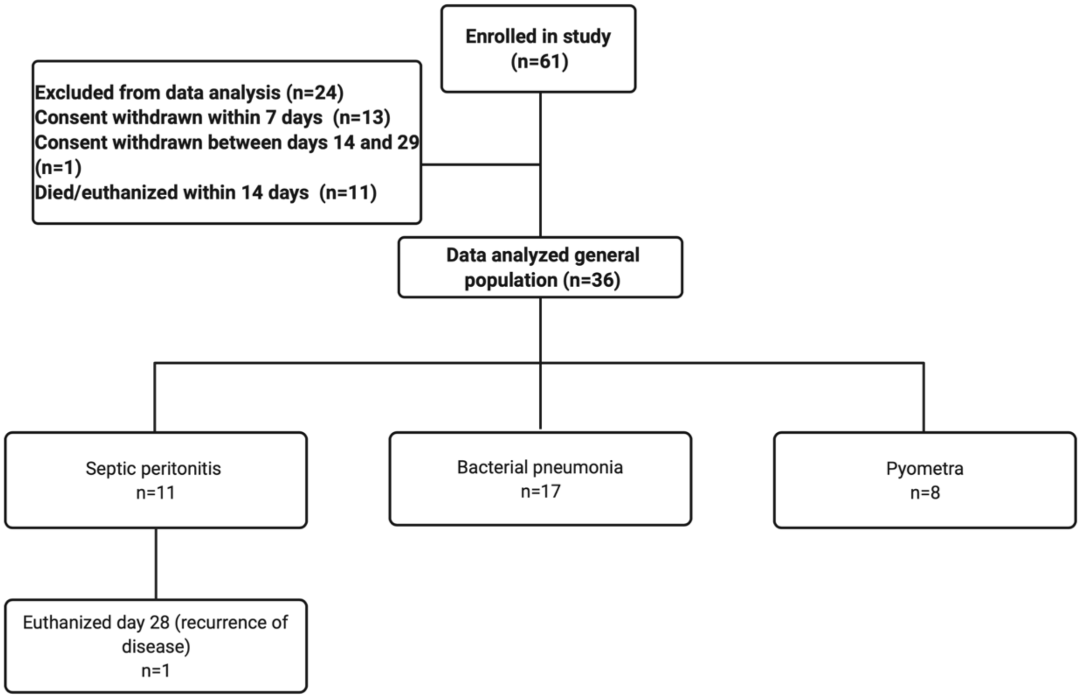

Figure S2: Fecal *Enterococcus faecalis* susceptibility over time. Proportion of dogs with MDR- *E. faecalis* at different time points are shown in panel A, with total proportion of dogs with any *E. faecalis* cultured in white, and dogs with MDR- *E. faecalis* shaded grey. Panels B through K depict susceptibility for the individual antimicrobial drugs tested. In those images, the white boxes also indicate the proportion of dogs with any *E. faecalis*, and the shaded boxes indicate the proportion of dogs with resistant *E. faecalis* to each respective drug as labeled. The statistically significant differences are denoted with symbols (*p ≤ 0.05; #p ≤ 0.01), and p-values as determined by Exact McNemar test are as follows: MDR difference between D1 and D7 0.0049; MDR difference between D1 and D14: 0.022; erythromycin D1 and D7: 0.004; minocycline D1 and D7: 0.0317; trimethoprim/sulfamethoxazole D1 and D14: 0.021; trimethoprim/sulfamethoxazole D1 and D7: 0.00.

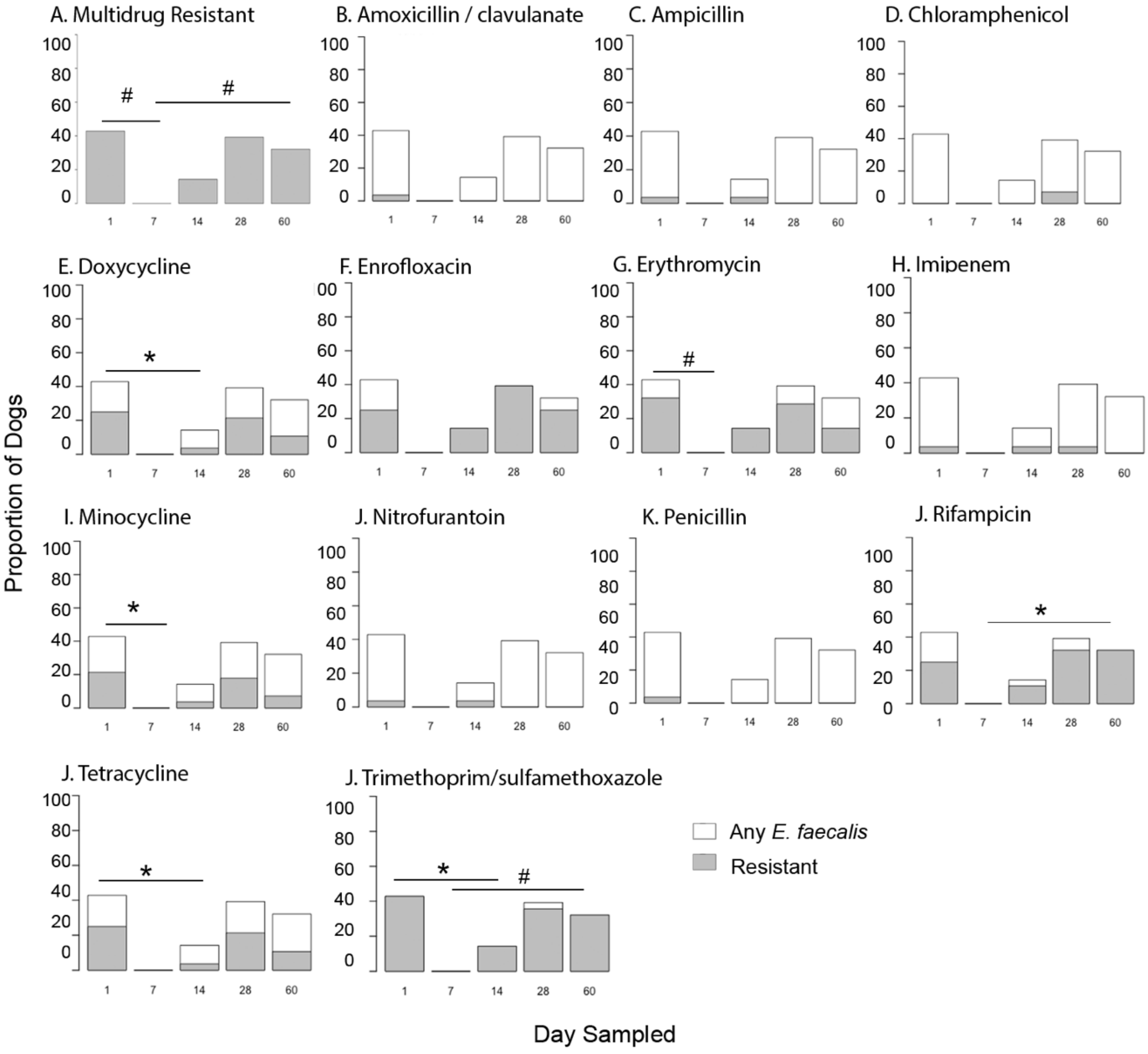

Figure S3: Fecal *Enterococcus faecium* susceptibility over time. Proportion of dogs with MDR-*E. faecium* at different time points are shown in panel A, with total proportion of dogs with any *E. faecium* cultured in white, and proportion of dogs with MDR- *E. faecium* shaded grey. Panels B through K depict susceptibility for the individual antimicrobial drugs tested. In those images, the white boxes also indicate the proportion of dogs with any *E. faecium*, and the shaded boxes indicate the proportion of dogs with resistant *E. faecium* to each respective drug as labeled. For MDR and resistance to individual drugs, with the exception of chloramphenicol, all D1 vs. D7 and D1 vs. D14 had significantly different recovery ($p \leq 0.001$). Day7 vs. D60 had $p \leq 0.002$ for all comparisons. *$p \leq 0.05$; #$p \leq 0.01$

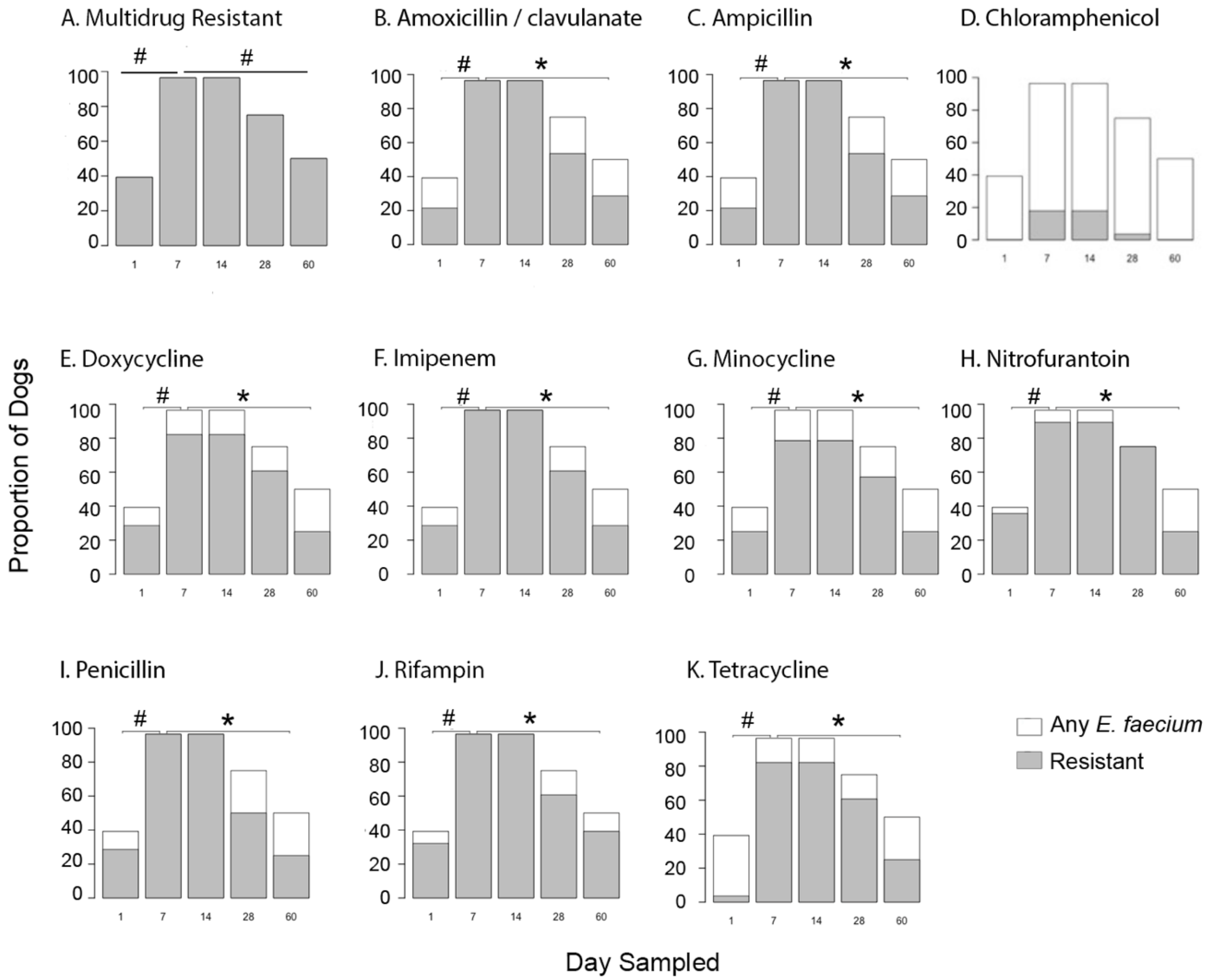

Figure S4: S Changes in fecal microbiota composition over the study period. Taxa are visualized by phylum (a) and class (b).

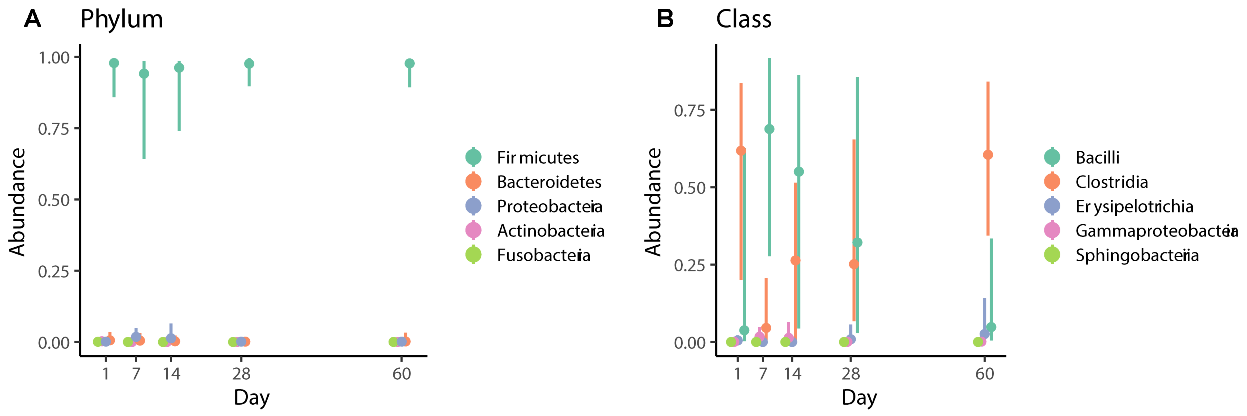

Supplement: Supplementary file 1 — Additional File 1. Figure S1. Patient enrollment summary. Figure S2. Fecal Enterococcus faecalis susceptibility over time. Figure S3. Fecal Enterococcus faecium susceptibility over time. Figure S4. Changes in fecal microbiota composition over the study period. [file 42523_2022_178_MOESM1_ESM.pdf]
